# Adherence to Insulin, Emotional Distress, and Trust in Physician Among Patients with Diabetes: A Cross-Sectional Study

**DOI:** 10.1007/s13300-018-0389-1

**Published:** 2018-03-08

**Authors:** Lucine Halepian, Mary Bou Saleh, Souheil Hallit, Lydia Rabbaa Khabbaz

**Affiliations:** 1 Pharmacology, Clinical Pharmacy and Drug Quality Control Laboratory, Saint-Joseph University of Beirut, Beirut, Lebanon; 2 Faculty of Pharmacy, Saint-Joseph University of Beirut, Beirut, Lebanon; 3 Faculty of Pharmacy, Lebanese University, Beirut, Lebanon; 4 Faculty of Medicine and Medical Sciences, Holy Spirit University of Kaslik, Kaslik, Lebanon; 5 Research Department, Psychiatric Hospital of the Cross, P.O. Box 60096, Jal Eddib, Lebanon; 6 Occupational Health Environment Research Team, U1219 BPH Bordeaux Population Health Research Center Inserm, Université de Bordeaux, Bordeaux, France; 7 INSPECT-LB: Institut National de Sante Publique, Epidemiologie Clinique et Toxicologie, Faculty of Public Health, Lebanese University, Beirut, Lebanon

**Keywords:** Adherence, Diabetes, Emotional distress, Insulin, Trust in physician

## Abstract

**Introduction:**

Type 2 diabetes represents a significant public health issue, with increasing prevalence in developing countries while adherence to insulin treatment remains a challenge. No studies have evaluated the relationship between adherence to insulin, diabetes-related distress, and trust in physician among persons with diabetes. Our objectives were to evaluate treatment adherence to insulin, emotional distress (using the Problem Areas in Diabetes Questionnaire, PAID), trust in physician, and to examine associations between them among Lebanese patients with diabetes.

**Methods:**

This cross-sectional study, conducted in all districts of Lebanon between August 2016 and April 2017, enrolled 135 adult patients.

**Results:**

The mean percentage score of adherence to insulin was 79.7 ± 19.94. A significantly higher mean adherence score was found in non-sedentary (81.96) compared to sedentary patients (67.41) (*p* = 0.017), with no difference between gender, employment, rural vs non-rural residence, or familial history of diabetes. In addition, no significant relationship was seen between adherence score and education level, smoking, or alcohol intake. A significant positive association was found between trust in physician and adherence scores, whereas a significant but negative one was found between PAID and adherence scores. The results of linear regressions showed that a secondary level of education (beta = − 13.48) significantly decreased the trust in physician score, whereas the total number of oral antidiabetics (beta = 0.93) increased it. Having a sedentary lifestyle (beta = − 12.73) and smoking < 3 waterpipes/week compared to no smoking (beta = − 16.82) significantly decreased the adherence score. Female gender (beta = 10.46), smoking < 3 waterpipes (beta = 27.42) and 3 + waterpipes/week (beta = 17.95) significantly increased the PAID score.

**Conclusion:**

Trust in physician is associated with an increased adherence and with decreased diabetes-related distress. This distress was also associated with poor adherence in our study.

**Electronic supplementary material:**

The online version of this article (doi:10.1007/s13300-018-0389-1) contains supplementary material, which is available to authorized users.

## Introduction

Every 6 s, a person dies in the world because of diabetes [1]. According to the latest report of the World Health Organization (WHO), type 2 diabetes (T2D) will be the seventh leading cause of death by 2030 [2]. Also, T2D presents a particularly high prevalence in both the Middle East and North Africa; among these regions, Lebanon occupies seventh place with a rate of 16.3% [1].

According to the WHO, adherence to treatment may be defined as the extent to which the patient’s history of therapeutic drug-taking coincides with the prescribed treatment [3]. Adherence to treatment remains a challenge in T2D since it is a chronic illness that is associated with a risk of comorbidity and requires a lifestyle change, especially after the onset of insulin therapy [4].

Even though insulin remains the most effective therapy to lower glycemia, the American Diabetes Association and the National Institute for Health and Clinical Excellence (NICE) guidelines recommend using insulin as a last resort, after several failures to achieve glycemic control with an oral glucose-lowering agent [5–7].

Low insulin adherence leads to poor glycemic control and may exacerbate microvascular and macrovascular complications such as kidney disease, dyslipidemia, stroke, retinopathy, nephropathy, and neuropathy and may increase episodes of hypoglycemia [8]. In fact, 45% of patients with T2D fail to achieve a good glycemic control [6].

Furthermore, according to several studies, diabetes may cause emotional distress which probably has an influence on medication adherence. Emotional distress is associated with depression, patient fears, and a lack of social and family support [9, 10].

In addition, trust in the healthcare provider is one of the central pillars of a patient–physician relationship. Previous studies conducted among adults with diabetes showed that increased trust in one’s physician has been associated with better glycemic control [11, 12]. The quality of information exchange and of primary care relationships are affected by the patient and provider communication behaviors [13]. In the short term, communicating with the patient can enhance trust in the physician, who will be able to incorporate treatment decisions according to the patient’s preferences and needs [13]. In addition, Schoenthaler et al. suggested that the quality of the patient–physician relationship has a strong influence on adherence to medication [14]. A better relationship contributes to the patients’ engagement in their care, which further influences outcomes related to self-care [15]. Conversely, when patients do not have confidence in their healthcare provider, they are less likely to implement care recommendations and thus to be adherent to their prescribed treatment [16].

Previous findings have suggested that high levels of diabetes-specific distress may account for higher functional impairment, work loss, and poorer self-management behavior [17–19]. Decreasing emotional distress is important in helping the patient with diabetes to cope with the disease and improving adherence to treatment [3]. Furthermore, Fisher et al. [20] identified seven major sources of distress among patients with type 1 diabetes, among them physician distress. This finding led us to hypothesize that trust may be a factor that contributes to physician-related diabetes distress, in type 1 as well as type 2 diabetes.

Several studies have been conducted to detect factors associated with insulin adherence worldwide [21–23], but none explored the impact of trust in physician and diabetes-related emotional distress on adherence to insulin. The primary objective of this study was to assess factors associated with insulin adherence among Lebanese patients with diabetes and to explore the relationship between this adherence, the patients’ trust in their healthcare provider, and diabetes-related emotional distress.

## Methods

### Study Design and Population

This cross-sectional study, conducted in all districts of Lebanon, was done between August 2016 and April 2017. Our sample was constructed from 20 community pharmacies selected randomly. Adult patients from any gender, with type 2 diabetes diagnosed at least 3 months ago, and treated with insulin or insulin analogues were eligible. Patients using an insulin pump were excluded.

### Data Collection

The questionnaire was distributed by trained interviewers. After obtaining the authorization from each pharmacy owner, the study objectives were explained to each patient after obtaining a written consent.

The self-administered anonymous questionnaire was in Arabic, the native language in Lebanon; it was composed of different sections: the first part included sociodemographic (age, gender, place of residence, marital status, and educational level), social habits (cigarette and waterpipe smoking, alcohol consumption), physical activity, family history of diabetes, duration and complications of diabetes. The second one comprised questions about insulin: name, dose, frequency of administration, shaking the vial or not before withdrawing, respecting the time of administration, storage, changing the needle after use, respecting the expiry date of the vial after opening; we also included questions about the level of patient’s fasting blood glucose (in the last checkup), the frequency of monitoring, the number of hypo- and/or hyperglycemia episodes during the last 12 months.

The scales used in this study (adherence, trust in physician, and PAID) were translated from English to Arabic through an initial translation and back-translation process. The English version was translated into Arabic by a certified translator, then this translation was translated again into English by another certified translator. Upon completion of this process, the translators compared the English versions of the scales to determine whether the variables had the same meaning. The back translation did not lead to any modification in the Arabic version of any scale.

### Adherence Assessment

The adherence to insulin was assessed using an adaptation of Lu et al.’s questionnaire [24] by asking the patients about the frequency, percentage, and rating response of their insulin use during the last month. Concerning the frequency, we asked the patient “did you take insulin all the time?” with the possible responses being divided as follow: 0% for none of the time, 20% for a little of the time, 40% for some of the time, 60% for a good bit of the time, 80% for most of the time, and 100% for all the time. The percentage item was checked using the question “what percent of the time were you able to take your medications exactly as your doctor prescribed them?” The rating item was assessed using the question “rate your ability to take all your medications as prescribed” with the possible answers being divided as follows: 0% = very poor, 20% = poor, 40% = fair, 60% = good, 80% = very good, and 100% = excellent. The total score was calculated by summing all three answers and presented in a percentage [24–26]. We calculated the reliability of each scale to assess the quality of our data. We obtained acceptable Cronbach alpha (0.621). Initially, the Lu et al.’s questionnaire was validated among patients with HIV and was later used in a population of patients attending general practice [26].

### Trust in Physician Scale

This scale is composed of 11 items (supplementary file), scored on a 5-point Likert scale ranging from 1 (strongly disagree) to 5 (strongly agree), except for questions 1, 5, 7, and 11 where the scoring was reversed. A summary measure of trust is obtained by taking the unweighted mean of the responses to the 11 questions and transforming that value to a 0–100 scale. Higher scores reflect greater trust. The wording of the 11 questions and the scoring system for each question are presented in Table 1. This scale was largely used among different participant populations and across a wide range of pathologies [27–30]. When completing the scale, respondents were instructed to think of the endocrinologist who usually provided their diabetes care [31]. We obtained high Cronbach alpha for this scale (0.892).

**Table 1:** Trust in physician scale questions and scoring

	Totally disagree	Disagree	Neutral	Agree	Totally agree
1. I doubt that my doctor really cares about me as a person	5	4	3	2	1
2. My doctor is usually considerate of my needs and puts them first	1	2	3	4	5
3. I trust my doctor so much I always try to follow his/her advice	1	2	3	4	5
4. If my doctor tells me something is so, then it must be true	1	2	3	4	5
5. I sometimes distrust my doctor’s opinions and would like a second one	5	4	3	2	1
6. I trust my doctor’s judgments about my medical care	1	2	3	4	5
7. I feel my doctor does not do everything he/she should about my medical care	5	4	3	2	1
8. I trust my doctor to put my medical needs above all other considerations when treating my medical problems	1	2	3	4	5
9. My doctor is well qualified to manage (diagnose and treat or make an appropriate referral) medical problems like mine	1	2	3	4	5
10. I trust my doctor to tell me if a mistake was made about my treatment	1	2	3	4	5
11. I sometimes worry that my doctor may not keep the information we discuss totally private	5	4	3	2	1

### Problem Areas in Diabetes Questionnaire (PAID)

The PAID measures diabetes-related emotional distress, which correlates with measures of related concepts such as depression, social support, health beliefs, and coping style, as well as predicts future blood glucose control of the patient [32]. The PAID is a self-report pencil and paper questionnaire that contains 20 items that describe negative emotions related to diabetes (e.g., fear, anger, frustration) commonly experienced by patients with diabetes. Completion takes approximately 5 min. Each question has five possible answers with a value from 0 to 4, with 0 representing “no problem” and 4 “a serious problem”. The scores are added up and multiplied by 1.25, generating a total score between 0–100. Patients scoring 40 or higher may be at the level of “diabetes-related distress” and warrant special attention. An extremely low score (0–10) combined with poor glycemic control may be indicative of denial. The Cronbach alpha for the PAID scale was excellent (0.909).

### Compliance with Ethical Guidelines

The study protocol was approved by the ethics committee of Saint-Joseph University of Beirut (reference number: USJ-2016-55). All procedures performed in studies involving human participants were in accordance with the ethical standards of the institutional research committee of Saint-Joseph University of Beirut and with the 1964 Declaration of Helsinki and its later amendments. Informed consent was obtained from all individual participants included in the study.

### Statistical Analysis

Data were collected and processed by Statistical Package for the Social Sciences SPSS, Version 23. Categorical variables were presented in frequencies and percentages, and continuous variables as means with standard deviations. Statistical analysis was conducted using Chi square, Fisher exact *t* test, and analysis of variance. ANOVA and Kruskal–Wallis tests were used to compare between three groups or more, and Pearson correlation coefficient was used to assess correlations between quantitative variables. In addition, a multivariate regression was conducted to reduce confounders. A linear regression was performed taking the adherence to treatment as the dependent variable. Variables which gave a* p* value less than 0.2 in the bivariate analysis were independent variables. A *p* value less than 0.05 was considered significant.

## Results

### Sociodemographic Characteristics of Patients

The total number of patients included in this study was 135 patients. The main sociodemographic characteristics of patients are presented in Table 2.

**Table 2:** Patients’ sociodemographic characteristics

Sex	
Male, *N* (%)	67 (49.6%)
Female, *N* (%)	68 (50.4%)
Age in years, mean (standard deviation)	65.84 (13.2)
Weight in kg, mean (standard deviation)	76.92 (16.1)
BMI (body mass index), mean (standard deviation)	27.66 (5.5)
Less than 18.5, *N* (%)	5 (3.7%)
Between 18.5 and 24.9, *N* (%)	36 (26.7%)
Between 25 and 29.9, *N* (%)	51 (37.8%)
30 or more, *N* (%)	43 (31.9%)
Marital status	
Married, *N* (%)	98 (72.6%)
Divorced/widower, *N* (%)	19 (14.1%)
Single, *N* (%)	18 (13.3%)
Sedentary lifestyle	
Yes, *N* (%)	21 (15.6%)
No, *N* (%)	114 (84.4%)
Residence	
Rural, *N* (%)	63 (46.7%)
Non rural, *N* (%)	72 (53.3%)
Working	
Yes, *N* (%)	41 (30.4%)
No, *N* (%)	94 (69.6)
Level of education	
Illiterate, *N* (%)	18 (13.3%)
Primary, *N* (%)	56 (41.5%)
Secondary, *N* (%)	26 (19.3%)
University, *N* (%)	35 (25.9%)
Cigarette consumption per day	
Non-smoker, *N* (%)	64 (47.4%)
1–14 cigarettes, *N* (%)	14 (10.4%)
15 or more cigarettes, *N* (%)	25 (18.5%)
Former smoker, *N* (%)	32 (23.7%)
Waterpipe consumption per week	
Non-smoker, *N* (%)	110 (81.5%)
Below 3, *N* (%)	7 (5.2%)
3 or more, *N* (%)	8 (5.9%)
Former smoker, *N* (%)	10 (7.4%)
Alcohol consumption per week	
Non-drinker, *N* (%)	94 (69.6%)
Up to one time, *N* (%)	26 (19.3%)
More than once, *N* (%)	9 (6.7%)
Former drinker, *N* (%)	6 (4.4%)

### Data Related to Clinical Characteristics of Diabetes

The mean duration of diabetes, since diagnosis, was 229.50 months (more than 19 years). Seventy-one percent of patients had a family history of diabetes and 88% were followed by a specialist in endocrinology. Ninety-three percent of patients in this study had comorbidities with an average of two concurrent medical conditions associated with diabetes. The results are shown in Table 3.

**Table 3:** Clinical characteristics of diabetes among participants

Diabetes duration in months, mean (standard deviation)	229.50 (126.02)
Family history of diabetes	
Yes, *N* (%)	96 (71.1%)
No, *N* (%)	39 (28.9%)
Physician’s visit frequency	
Mean number of visits/year (standard deviation)	4.59 (5.14)
Comorbidities, mean (standard deviation)	6.00 (2.95)
0 comorbidity, number of patients (%)	6 (4.4%)
1 comorbidity	3 (2.2%)
2 comorbidities	7 (5.2%)
3 comorbidities	9 (6.7%)
4 comorbidities	13 (9.6%)
5–9 comorbidities	81 (60.0%)
10+ comorbidities	16 (11.7%)

### Data Related to Insulin Therapy

Information regarding insulin therapy is summarized in Table 4. Six different patterns of insulin administration were observed. Most patients, about 76%, used the regimen based on the administration of a basal insulin alone or a basal insulin and bolus premix. The remaining patients were on other regimens. The majority of patients (79%) used insulin pens.

**Table 4:** Diabetes treatment

Duration of therapy in months, mean (standard deviation)	85.74 (89.47)
Total daily dose in IU/kg, mean (standard deviation)	0.49 (0.33)
Delivery device	
Pen, *N* (%)	107 (79.3%)
Vial, *N* (%)	27 (20.0%)
Pen and vial, *N* (%)	1 (0.7%)
Types of insulin	
One, *N* (%)	106 (78.5%)
Two, *N* (%)	28 (20.75%)
Three, *N* (%)	1 (0.75%)
Number of insulin injection per day, mean (standard deviation)	2.63 (1.69)
Concomitant use of oral antidiabetic drugs	
Yes, *N* (%)	94 (69.6%)
No, *N* (%)	41 (30.4%)
Number of oral antidiabetic drugs/participant, mean (standard deviation)	3.87 (2.18)

Thirteen percent of the patients were well controlled with an HbA1C < 7%. Forty-nine percent were considered to be reasonably controlled with an HbA1C between 7% and 8%. Thirty-five percent were poorly controlled (HbA1c > 8%).

Concerning the patient’s education, 88% of the patients gave a correct answer when asked about the normal range of fasting blood glucose and 84% knew the target levels of HbA1C that provide a good diabetes control (HBA1C < 8%). Furthermore, 63% of these patients were practicing a self-glucose monitoring test with an average frequency of once a day.

### Average Scores Obtained for Trust in Physician Scale, PAID, and Adherence to Insulin

The mean trust in physician score was 73.35 ± 20.38 (median = 75), whereas the mean PAID and adherence scores were 32.75 ± 29.37 (median = 22.41) and 79.7 ± 19.94 (median = 87.5), respectively.

### Bivariate Analysis

A significantly higher mean adherence score was found in non-sedentary (81.96) compared to sedentary patients (67.41). A significantly higher PAID score was found in female compared to male patients (37.27 vs 28.11), in sedentary compared to non-sedentary patients (42.38 vs 30.96). A significantly higher mean trust in physician score was found in illiterate patients (78.66) compared to other levels of education, and in those who smoked below three waterpipes weekly (55.35) compared to other groups (Table 5).

**Table 5:** Bivariate analysis of factors associated with the adherence to insulin, trust in physician, and PAID scores

Variable	Trust in physician score	PAID score	Adherence score
Gender			
Male	71.43 ± 19.58	28.11 ± 22.75*	78.40 ± 20.66
Female	75.23 ± 21.11	37.27 ± 21.27*	80.97 ± 19.28
Sedentary lifestyle			
No	74.28 ± 20.67	30.96 ± 21.53*	81.96 ± 18.16*
Yes	68.29 ± 18.33	42.38 ± 25.04*	67.41 ± 24.77*
Residence			
Urban	74.77 ± 19.17	30.44 ± 20.30	81.90 ± 15.85
Rural	71.71 ± 21.71	35.35 ± 24.47	77.18 ± 23.66
Working status			
No	74.08 ± 20.35	32.24 ± 21.93	79.52 ± 20.57
Yes	71.67 ± 20.59	33.96 ± 23.72	80.10 ± 18.65
Family history of diabetes			
No	73.83 ± 21.83	29.71 ± 24.12	84.77 ± 16.19**
Yes	73.15 ± 19.87	34.01 ± 21.67	77.63 ± 21.01**
Concomitant use of oral antidiabetics			
No	73.66 ± 19.11	33.44 ± 23.12	78.96 ± 16.88
Yes	73.21 ± 21.01	32.45 ± 22.21	80.02 ± 21.22
Marital status			
Married	73.49 ± 21.41	30.95 ± 20.98	80.48 ± 19.43
Divorced/widowed	78.11 ± 17.77	38.75 ± 25.97	78.12 ± 26.25
Single	67.55 ± 16.19	36.47 ± 25.83	77.08 ± 15.42
Educational level			
Illiterate	78.66 ± 20.91*	33.05 ± 26.05	73.26 ± 28.80**
Primary	77.59 ± 20.16*	30.65 ± 19.54	82.75 ± 17.58**
Secondary	62.50 ± 19.60*	40.52 ± 25.45	74.51 ± 18.38**
University	71.88 ± 18.46*	30.11 ± 22.02	81.96 ± 18.47**
Cigarette consumption per day			
Non-smoker	74.89 ± 18.83	37.01 ± 23.94**	79.63 ± 20.54
1–14 cigarettes	67.04 ± 27.97	31.69 ± 24.21**	80.35 ± 16.95
15+ cigarettes	71.72 ± 22.59	25.45 ± 21.59**	76.37 ± 22.50
Former smoker	74.28 ± 18.03	30.54 ± 17.76**	82.12 ± 18.29
Waterpipe consumption per week			
Non-smoker	74.17 ± 20.33	30.67 ± 21.67*	80.62 ± 18.79**
Below 3	59.74 ± 25.02	55.35 ± 21.49*	60.26 ± 33.20**
3 or more	74.14 ± 21.89	48.12 ± 20.53*	81.64 ± 23.17**
Former smoker	73.18 ± 15.29	27.37 ± 21.35*	81.56 ± 13.53**
Weekly alcohol consumption			
Non-drinker	73.74 ± 21.08	33.24 ± 21.73	81.51 ± 19.08
Up to one time	74.91 ± 18.87	31.34 ± 24.91	75.72 ± 21.51
More than one time	64.89 ± 22.87	36.66 ± 26.85	72.57 ± 24.98
Former drinker	73.10 ± 9.89	25.41 ± 17.35	79.16 ± 18.39
Total daily dose of insulin			
Delivery device			
Pen	73.49 ± 19.83	32.96 ± 22.11	80.22 ± 18.76
Vial	71.88 ± 22.63	30.74 ± 23.42	77.54 ± 24.69
Both	97.72 ± 0.00	65.00 ± 0.00	81.25 ± 0.00
Types of insulin			
One	73.24 ± 20.72	33.23 ± 22.36	78.98 ± 21.24
Two	72.88 ± 19.23	29.67 ± 22.33	82.36 ± 14.51
Three	97.72 ± 0.00	65.00 ± 0.00	81.25 ± 0.00

*p < 0.05; **p > 0.05 but < 0.2

Furthermore, a significant negative correlation was found between the trust in physician score and the PAID score (*r* = − 0.289) and a positive one with the adherence score (*r* = 0.416). A significant negative correlation was found between the PAID score and the adherence score (*r* = − 0.3), duration of diabetes since diagnosis (*r* = − 0.172), and the duration of insulin therapy (*r* = − 0.239) (Table 6).

**Table 6:** Correlation coefficients between the scores and continuous variables

	Trust in physician score	PAID score	Adherence score
Trust in physician score	1	− 0.289*	0.416*
PAID score	− 0.289*	1	− 0.300*
Adherence score	0.416*	− 0.300*	1
Age	0.078	− 0.071	− 0.044
BMI	0.009	0.076	− 0.017
Duration of diabetes diagnosis	0.028	− 0.172*	0.047
Frequency of physician’s visits	0.086	− 0.031	0.081
Number of comorbidities	0.048	− 0.104	− 0.068
Duration of insulin therapy	0.133**	− 0.239*	0.000
Total insulin daily dose	− 0.079	0.144	0.003
Total daily number of injections	− 0.008	0.020	0.058
Total number of oral antidiabetics	0.167**	− 0.123**	0.137**

*p < 0.05; **p > 0.05 but < 0.2

### Multivariable Analysis

The results of a first stepwise linear regression, taking the trust in physician score as the dependent variable, showed that a secondary level of education was significantly associated with a decrease in the trust in physician score (beta = − 13.48), whereas the total number of oral antidiabetics was significantly associated with an increase in that score (beta = 0.93).

A second stepwise linear regression, taking the adherence score as the dependent variable, showed that having a sedentary lifestyle (beta = − 12.739) and smoking less than three waterpipes per week compared to no waterpipe smoking (beta = − 16.826) were significantly associated with a decrease in the adherence score.

A third stepwise linear regression, taking the PAID score as the dependent variable, showed that female gender (beta = 10.467), smoking less than three waterpipes (beta = 27.426), and three or more waterpipes weekly (beta = 17.951) compared to no waterpipe smoking were significantly associated with an increase in the PAID score (Table 7).

**Table 7:** Multivariable analyses

Variable	Unstandardized beta	Standardized beta	*p* value	Confidence interval	
**Linear regression 1 taking the trust in physician score as the dependent variable**					
Secondary level of education (compared to illiteracy)	− 13.486	− 0.262	0.002	− 21.913	− 5.059
Total number of oral antidiabetics	0.934	0.169	0.043	0.028	1.840
**Linear regression 2 taking the adherence score as the dependent variable**					
Sedentary lifestyle	− 12.739	− 0.232	0.006	− 21.814	− 3.663
Less than 3 waterpipes per week compared to no waterpipe smoking	− 16.826	− 0.188	0.027	− 31.660	− 1.991
**Linear regression 3 taking the PAID score as the dependent variable**					
Less than 3 waterpipes per week compared to no waterpipe smoking	27.426	0.273	0.001	11.221	43.632
Gender	10.467	0.234	0.005	3.267	17.667
3 or more waterpipes per week compared to no waterpipe smoking	17.951	0.191	0.020	2.815	33.087

## Discussion

To the best of our knowledge, our study is the first to assess the adherence of patients with diabetes type 2 to their insulin treatment and the factors associated with this adherence, in Lebanon. Adherence to treatment is a key element in the management of diabetes to firstly ensure good glycemic control and also to reduce or delay the onset of microvascular and macrovascular complications of diabetes.

Our study showed a highly significant association between the degree of patients’ trust in their doctor and treatment adherence. The greater the degree of confidence, the greater adherence to insulin. This result is in agreement with the study by White et al. (2013) which showed that adherence to therapy is directly proportional to the degree of patient trust in the doctor [12]. In a cross-sectional diabetes study, older patients’ evaluations of how well their physicians provided information on their illness and treatment were associated with patient self-reported medication-taking behaviors [33]. A study in the Kaiser Permanente population found that a greater proportion of patients who failed to initiate insulin felt that their healthcare providers inadequately explained the risks and benefits of insulin, compared to those who initiated insulin [34]. More patient counseling by healthcare professionals is needed to educate the patient about the possible side effects that might occur with insulin treatment and most importantly about the efficacy of insulin [34, 35]. Emphasizing the advantages of insulin treatment at the time of prescribing, as well as explaining the probability of an adverse side effect happening and its seriousness, may improve how patients receive information from other sources.

Our results demonstrated that the degree of patients’ trust in their physician was negatively associated with the diabetes-related distress, in line with previous findings [36] that showed that the physician effective communication behavior and a higher trust in him, as reported by the patient, were inversely related to emotional distress among patients with diabetes [37]. Some interventions might be used to increase the patient’s trust in the physician; these include training interventions to improve physician behavior or providing the patients with more information about their treatment and giving them a chance to discuss options [38].

Furthermore, this study showed a significant negative association between adherence to insulin and the patient’s distress. Emotional distress was associated with poor adherence which is consistent with previous studies [39, 40]. Negative, “blocking” thoughts adversely impact the persons’ self-care behavior, thereby unintentionally reinforcing a negative cycle of events, which can ultimately lead to a condition referred to by Polonsky as “diabetes burnout” [41]. Emotional and social support were shown to increase medication adherence in diabetes [42] by increasing the patient’s active coping behavior [43]. Stronger social support from family, friends, and communities could cultivate positive mental and emotional changes. It strengthens patients’ resolve, belief, and confidence in managing their condition, and improves their quality of life [44].

Inactivity was found in our study to decrease the adherence to insulin score, in agreement with previous studies [45]. Physical activity plays a vital role in the self-management of T2DM and exercise is the best predictor of maintaining weight, and can decrease insulin resistance [46]. Fear of hypoglycemia is one of the factors that could prevent the patient from performing any physical activity. In the present study, participants with a higher awareness of hypoglycemia risk were more prone to reduce their physical activity. This is consistent with the finding of the study done in Scotland which stated that main reason for inactivity is due to fear of hypoglycemia [47].

A secondary level of education was negatively associated with a decrease in the trust in physician score. This might be explained by the fact that patients with higher educational attainment are more involved in the medical decision-making process and verify the credibility of information provided by their physician and sometimes explore options beyond what is given during their medical visit [48]. People with a lower literacy level may be more willing to trust their physician and accept their recommendations without questioning them.

Smoking waterpipe was negatively associated with adherence to treatment but positively associated with the diabetes-related distress, in line with the study by Solberg et al. [49] who found that smokers with diabetes report more sadness or depression compared to non-smokers with diabetes [49]. Waterpipe smoking being associated with a decrease in adherence to treatment is similar to the findings of Ciechanowski et al. [50] but opposite to those of Solberg et al. [49].

This study also showed that patients who do not have a family history of diabetes were more adherent to insulin than patients with a family history of diabetes, in agreement with the findings of Riaz et al. [45]. This can be attributed to the higher vigilance of these patients about their disease and the fear of future complications and their impact on their quality of life. On the other hand, our study showed no association between adherence and gender, age, diabetes duration since diagnosis, and the patient’s level of education, consistent with several previous studies [23, 51–53].

### Limitations

Our study has a number of limitations. The total sample size is small and might not be representative of the whole population. This is a cross-sectional self-designed survey with retrospective reports, and consequently a low level of evidence. The effect of the recall bias could be differential and lead to the overestimation of effects for some known risk factors. An information bias is also possible since the use of a questionnaire may not always be accurate: problems in question understanding, recall deficiency, and over- or underevaluating symptoms may still be possible. In addition, the cost of medications, a factor known to affect adherence, was not taken into consideration even though we think that it may not have been a strong bias in this study because 88% of the patients had either private insurance or social security paying for their medications.

## Conclusion

Patient adherence to insulin is an important criterion for achieving the desired therapeutic outcomes in diabetes management. Distress in diabetes shows a negative association with adherence. Good healthcare professional–patient communication is a major expectation and an important parameter improving drug adherence. It determines the patients’ trust in their physician, thus improving adherence.

## Electronic supplementary material

Below is the link to the electronic supplementary material.

Supplementary material 1 (DOCX 17 kb)
