# Supplementary material for: Adherence to Insulin, Emotional Distress, and Trust in Physician Among Patients with Diabetes: A Cross-Sectional Study
Source: Diabetes Ther. 2018 Mar 8;9(2):713–26. doi: 10.1007/s13300-018-0389-1 (PMC6104261; doi:10.1007/s13300-018-0389-1)
Supplement: Supplementary file 1 — Supplementary material 1 (DOCX 17 kb) [file 13300_2018_389_MOESM1_ESM.docx]

**Trust in Physician Scale**

|  | **Totally disagree** | **Disagree** | **Neutral** | **Agree** | **Totally agree** |
| --- | --- | --- | --- | --- | --- |
| 1 .I doubt that my doctor really cares about me as a person. | **5** | **4** | **3** | **2** | **1** |
| 2. My doctor is usually considerate of my needs and puts them first. | **1** | **2** | **3** | **4** | **5** |
| 3. I trust my doctor so much I always try to follow his/her advice. | **1** | **2** | **3** | **4** | **5** |
| 4. If my doctor tells me something is so, then it must be true. | **1** | **2** | **3** | **4** | **5** |
| 5. I sometimes distrust my doctor’s opinions and would like a second one. | **5** | **4** | **3** | **2** | **1** |
| 6. I trust my doctor’s judgments about my medical care. | **1** | **2** | **3** | **4** | **5** |
| 7. I feel my doctor does not do everything he/she should about my medical care. | **5** | **4** | **3** | **2** | **1** |
| 8. I trust my doctor to put my medical needs above all other considerations when treating my medical problems. | **1** | **2** | **3** | **4** | **5** |
| 9. My doctor is well qualified to manage (diagnose and treat or make an appropriate referral) medical problems like mine. | **1** | **2** | **3** | **4** | **5** |
| 10. I trust my doctor to tell me if a mistake was made about my treatment. | **1** | **2** | **3** | **4** | **5** |
| 11. I sometimes worry that my doctor may not keep the information we discuss totally private. | **5** | **4** | **3** | **2** | 1 |
